# Mechanical Models of the Dynamics of Vitreous Substitutes

**DOI:** 10.1155/2014/672926

**Published:** 2014-07-24

**Authors:** Krystyna Isakova, Jan O. Pralits, Rodolfo Repetto, Mario R. Romano

**Affiliations:** ^1^Department of Civil, Chemical and Environmental Engineering, University of Genoa, 16145 Genoa, Italy; ^2^Department of Neuroscience, University of Naples Federico II, 80131 Naples, Italy; ^3^Humanitas Clinical and Research Center, 20089 Milan, Italy

## Abstract

We discuss some aspects of the fluid dynamics of vitreous substitutes in the vitreous chamber, focussing on the flow induced by rotations of the eye bulb. We use simple, yet not trivial, theoretical models to highlight mechanical concepts that are relevant to understand the dynamics of vitreous substitutes and also to identify ideal properties for vitreous replacement fluids. We first recall results by previous authors, showing that the maximum shear stress on the retina grows with increasing viscosity of the fluid up to a saturation value. We then investigate how the wall shear stress changes if a thin layer of aqueous humour is present in the vitreous chamber, separating the retina from the vitreous replacement fluid. The theoretical predictions show that the existence of a thin layer of aqueous is sufficient to substantially decrease the shear stress on the retina. We finally discuss a theoretical model that predicts the stability conditions of the interface between the aqueous and a vitreous substitute. We discuss the implications of this model to understand the mechanisms leading to the formation of emulsion in the vitreous chamber, showing that instability of the interface is possible in a range of parameters relevant for the human eye.

## 1. Introduction

Retinal detachment is a serious, sight threatening condition that occurs when fluid enters the potential space between the neurosensory retina and the retinal pigment epithelium. Posterior vitreous detachment is primarily responsible for the generation of tractions on the retina that might produce retinal tears. These can possibly evolve into retinal detachment, since the detached vitreous often displays tight attachment points with the retina, where concentrated mechanical stimuli occur [1]. In the general population, nontraumatic phakic rhegmatogenous retinal detachment occurs in about 5.4 out of 100,000 persons and is among the most frequent causes of blindness in Western countries [2].

Surgery is the only viable way to treat retinal detachment [3]. One of the most common surgical treatments consists in removing the vitreous gel from the eye, peeling epiretinal traction, flattening the retinal detachment and closing retinal tears, and inducing chorioretinal adhesion. Materials that form an interface with the aqueous environment of the eye can be effective in closing retinal breaks and holding the retina in place against the retinal pigment epithelium. They are called vitreous substitutes or tamponade fluids.

Various vitreous substitutes are employed in the surgical practice, with largely different mechanical properties [4, 5]. In particular, artificial vitreous substitutes can be classified into three categories: gases, liquids, and gels. Polymetric hydrogels are only used as a support for sustained drug delivery in the vitreous. Currently, the most commonly used fluids employed as vitreous substitutes are gases, silicone oils, perfluorocarbon liquids, and semifluorinated liquids. Gases and perfluorocarbon liquids are used as short-term substitutes, especially during intraoperative procedures. Semifluorinated liquids, owing to their toxicity, are also only used as short-term vitreous substitutes.

At present, the only long-term vitreous substitutes widely employed in the clinical practice are silicone oils. They have suitable properties of chemical stability and transparency and have a high surface tension with the aqueous humour, which is a desirable property. The rational of using silicone oil as intraocular tamponade is to interrupt the open communication between the subretinal space/retinal pigment epithelial cells and the preretinal space with the aim of securing, in the first few days after surgery, chorioretinal adhesion induced by cryo- or laser treatment. Depending on the location of the retinal break oils with different densities (either higher or lower than the aqueous) can be adopted [6, 7]. Proper patient posture is required after the injection, in order to maintain the contact of the tamponade with the retinal break. Direct contact between the tamponade fluid and retina is indeed difficult to determine. Due to the oil hydrophobicity a thin layer of aqueous is likely to form between the retina and vitreous substitute. This is irrelevant where the retina is attached to the pigment epithelium but is crucial in correspondence with the break. It has been shown theoretically and experimentally that, the supported area of the retina is strongly affected by the contact angle between the oil and the retina [8].

The mechanical properties of tamponade fluids (density, viscosity, and surface tension with the aqueous) influence the efficiency of the treatment and, therefore, a full understanding of the mechanical implications associated with the surgery is desirable. With the present work we aim at clarifying, from a purely mechanical point of view, the implications of adopting tamponade fluids with different mechanical properties. The problem is extremely complex even if only mechanics is accounted for, and, therefore, we proceed in this paper by introducing simple theoretical models that shed some light on specific, yet crucial, aspects of the problem.

We start by considering the effect of viscosity of the tamponade fluid on the mechanical actions exerted on the retina during eye rotations.

Due to the limited tamponade effect of silicone oils we then investigate further factors leading to the successful surgery. In particular, we investigate the changes of the maximum wall shear stress when silicone oils are used, accounting for the possible presence of a thin layer of aqueous separating the retina from the tamponade fluid.

The success rate of surgery when silicone oils are used is about 70%. One of the common problems after vitrectomy, especially in the long run, is the formation of an oil emulsion. The reasons why this happens when silicone oils are used as tamponades are still unclear. A further aim of this paper is to present a simple theoretical model that predicts the role of oil properties (particularly, viscosity and surface tension) in the process of emulsion formation. To this end we study the stability of the interface between two superposed immiscible fluids set in motion by movements of the eye.

## 2. Materials and Methods

The results presented in this paper are based on solutions of the mathematical equations that govern the motion of fluids. Fluid dynamics is a very well developed branch of physics, the modern foundations of which date back to the 19th century. The so-called Navier-Stokes equations, named after Claude-Louis Navier and George Gabriel Stokes, are known to accurately model the motion of a viscous fluid described as a continuum body. These equations are mathematically very complex and admit closed-form solutions, that is, solutions that can be expressed analytically in terms of known functions, only in very special cases. If an analytical solution of a problem can be found, its dependency on the controlling parameters (e.g., in the present case the size of the vitreous chamber, the viscosity of the fluid, and so forth) can be easily determined, without the need of computational simulations, and physical insight on the problem is therefore effectively obtained. In this paper we discuss some analytical solutions of the Navier-Stokes equations, which are relevant to understanding the dynamics of vitreous substitutes.

We consider purely viscous fluids, that is, fluids whose mechanical properties are completely characterized by the density *ρ* (mass per unit volume) and the (dynamic) viscosity *μ* (which is a measure of resistance to flow) and in which the stress is linearly proportional to the rate of deformation. Water, aqueous humour, and oils fall into this category.

Fluid motion in the vitreous chamber can be driven by different mechanisms, in particular, rotations of the eyeball or thermal differences between the anterior and posterior segments of the eye. However, it can be shown by simple order-of-magnitude arguments that the motion induced by eye rotations is much stronger than the thermally driven flow [9] and, therefore, we restrict our attention to the former. Eye rotations induce motion in the fluid contained in the eye owing to the so-called no-slip boundary condition, according to which fluid particles in contact with a rigid wall (e.g., the vitreous chamber wall) move at the same velocity as the wall itself. In other words, fluid particles do not flow across the wall and they do not slip over it.

We consider three different, relatively simple, models that shed light on important aspects of the dynamics of vitreous substitutes in the vitreous chamber. Proper interpretation of results from experimental or more complex theoretical models requires a full understanding of the results presented here. The details of the mathematical models are briefly reported in the appendices.

**Figure 1:**
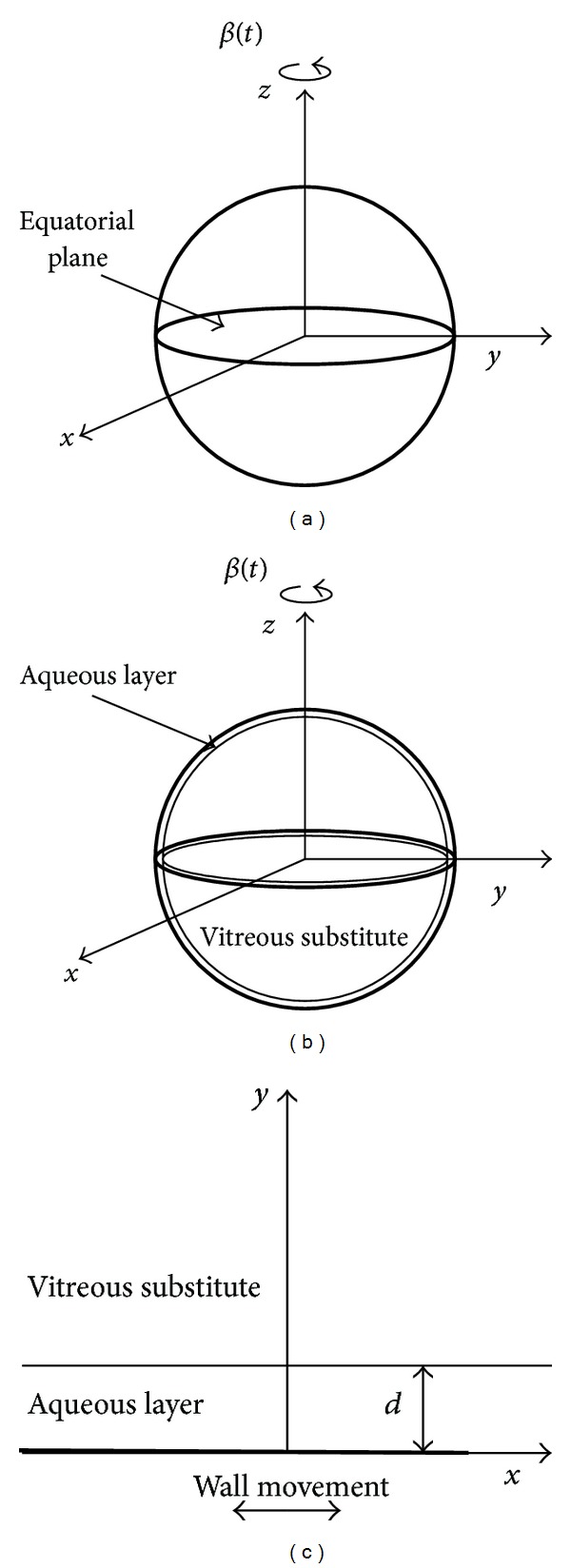
Sketch of the three models adopted in the paper.

*Model 1.* We first review results obtained by previous authors concerning the case of a rigid hollow sphere of radius *R*, modelling the vitreous chamber, filled with a fluid and study fluid motion generated by small-amplitude, periodic, torsional oscillations of the sphere (see Figure 1(a)). This problem has been studied in [10, 11] for the case of viscoelastic fluids. In reality, the vitreous chamber is not perfectly spherical, particularly owing to the indentation produced in its anterior part by the lens. The effect of departure from the spherical shape on fluid motion has been studied theoretically and experimentally by several authors [12–16]; however, for the present purposes and for the sake of the simplicity, it is sufficient here to consider a spherical shape. Fluid motion generates stresses on the wall, which we determine analytically. We discuss the qualitative characteristics of the flow and show the dependency of the stress at the wall on fluid viscosity.

*Model 2.* We then investigate how the stress on the wall is modified when a second fluid is present within the domain (see Figure 1(b)). This typically happens when a hydrophobic vitreous substitute, such as silicon oil, is injected into the vitreous chamber: a thin layer of aqueous close to the wall separates the vitreous substitute from the retina. In order to model this condition we adopt an idealized geometry consisting of a rigid sphere filled with two immiscible fluids (aqueous and vitreous substitute) arranged concentrically, with the aqueous in the external layer. In other words we assume that the thickness *d* of the aqueous is uniform. This allows us to solve the problem for the motion of the two fluids analytically. We then compute the wall shear stress on the equatorial plane.

*Model 3.* Finally, we study the stability of the interface between the aqueous layer and the vitreous substitute, when the two fluids are set in motion by eye rotations. For the sake of simplicity we assume that the thickness of the aqueous layer is much smaller than the eye radius, which is often a realistic assumption, and, as a first approach to the problem, we neglect the curvature of the retinal surface and consider a flat wall (see Figure 1(c)). The configuration of the interface between the two fluids is assumed to be perturbed by small (formally infinitesimal) sinusoidal waves (normal mode analysis) and we study whether the amplitude of these disturbances grows or decays in time. In the former case we infer instability of the system, and in the latter we infer stability. Some details of the mathematical analysis, which is quite technical, are given in the appendices. Instability of the interface may be considered as a possible incipient condition leading to the breakdown of the interface and can, therefore, represent a route towards emulsification. We note that the model is based on a so-called linear stability analysis: this allows us to establish whether perturbations will grow or decay in time (the model actually predicts exponential growth or decay), providing a threshold value for the onset of instability. The model allows us to establish how the interface stability conditions depend on the properties of the vitreous substitute, particularly, its surface tension with the aqueous and its viscosity.

## 3. Results and Discussion

### 3.1. Wall Shear Stress in a Periodically Rotating Sphere

**Figure 2:**
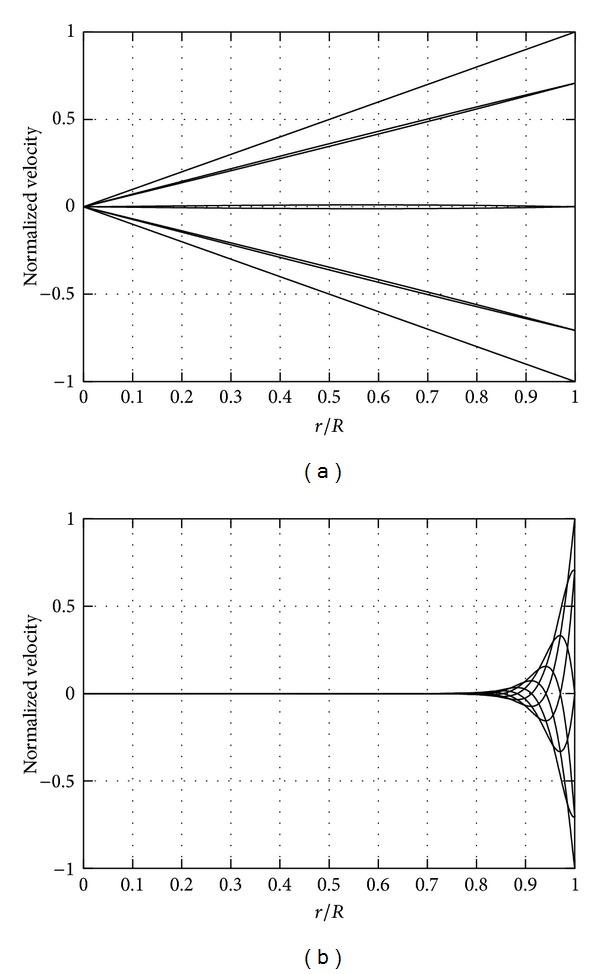
Velocity profiles in radial direction. *r* = 0 corresponds to the centre of the sphere and *r* = 1 corresponds to the location of the wall. The velocity is normalized with the maximum velocity at the wall. In both figures we assumed that the sphere contains purely viscous fluids and that the frequency of rotations is equal to 20 rad/s. (a) Silicon oil, *μ* = 0.96 Pa*·*s; (b) water, *μ* = 0.001 Pa*·*s.

We first consider the motion of a fluid contained in a sphere of radius *R*, performing periodic rotations of amplitude *A* and frequency *ω*. If the rotation amplitude *A* is small, it can be shown that, at leading order, the fluid velocity vectors are everywhere orthogonal to the axis of rotation [10, 11]. In other words, the velocity has only the azimuthal component. Moreover, the velocity oscillates with the same frequency as the sphere rotations. In Figures 2(a) and 2(b) we plot velocity profiles attained in a viscous fluid on the equatorial plane orthogonal to the axis of rotation. We note that this is the plane where the stress on the wall attains its maximum value. In the figure we show the variation of the azimuthal velocity in the radial direction and each curve corresponds to a different time within the period. The velocity is zero at the centre of the domain (*r* = 0) and has the same velocity of the wall at *r* = *R*. In the two cases the frequency is kept constant and is equal to 20 rad/s, which is a realistic value for real eye rotations. In Figure 2(a) we use a viscosity typical of a silicon oil (*μ* = 0.96 Pa*·*s [17]), whereas Figure 2(b) is obtained assuming the viscosity of water (*μ* = 0.001 Pa*·*s). In the two cases the velocity profiles are significantly different. In the high viscosity case they are almost straight lines; in other words the fluid moves almost as if it was a rigid body. On the other hand, when the viscosity is small, a thin layer forms at the wall in which the fluid moves and the velocity in the core of the domain is vanishingly small. This layer is referred to as an oscillatory boundary layer. The thickness of the oscillatory boundary layer at the wall is of order *δ* ~ √(*μ*/*ρω*). This means that similar results could have been obtained by keeping fixed the viscosity of the fluid and changing the frequency of oscillations. In fact, the problem is governed by a single dimensionless parameter *α*, the Womersley number, defined as *α* = √((*ρR* ^2^ *ω*)/*μ*), which can be physically interpreted as the ratio *R*/*δ*, between the radius of the sphere and the thickness of the oscillatory boundary layer. Flows characterized by the same value of the Womersley number have identical velocity profiles.

In purely viscous fluids, whatever the value of the viscosity, the maximum of the velocity is invariably attained at the wall (*r* = *R*). We note that the real healthy vitreous is a viscoelastic fluid [18, 19], that is, a fluid in which the state of stress depends on the history of deformation. In other words viscoelastic fluids have a “fading” memory.  Figure 6 in the paper by Meskauskas et al. [11] is the equivalent of Figure 2 of the present paper but is obtained taking into account the viscoelasticity of the fluid and adopting values of the vitreous properties obtained in [19] from ex vivo experiments on porcine eyes. The velocity profiles show striking qualitative differences with respect to those obtained for purely viscous fluids (Figure 2 of this paper). In particular, in the case of a viscoelastic fluid, the maximum velocity can be attained in the core of the domain and not at the wall. This phenomenon is due to a resonant excitation of vitreous motion. When resonance occurs, large values of the stress are attained on the boundary of the domain, that is, on the retina.

**Figure 3:**
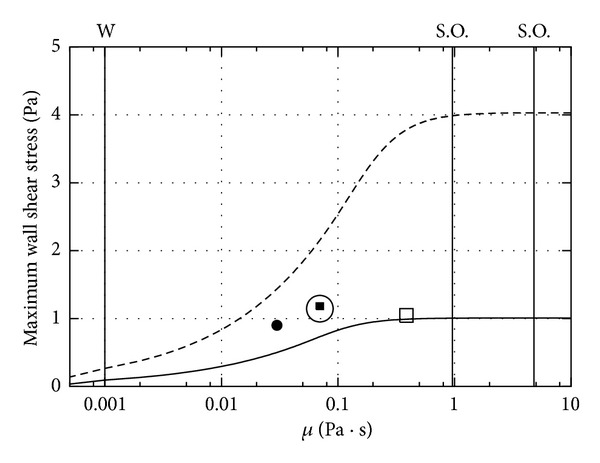
Dependency of the maximum shear stress at the wall on the viscosity in the case of a purely viscous fluid. The two curves correspond to two different values of the frequency of eye rotations (dashed line 20 rad/s; solid line 10 rad/s; A = 20 deg = *π*/9 rad). W: water; S.O.: silicon oils (*ρ* = 960 Kg/m^3^, *μ* = 0.96 Pa*·*s, and *μ* = 4.8 Pa*·*s). In the figure we also report with symbols the values of the maximum wall shear stress obtained in the case of a viscoelastic fluid and adopt the rheological properties measured in [18, 19]. Solid square: complex viscosity μ* = 0.39 − *i* Pa*·*s, *ω* = 10 rad/s [18]; empty square: μ* = 0.07 − 0.28*i* Pa*·*s, *ω* = 10 rad/s [18]; solid circle: μ* = 0.07–0.28*i* Pa*·*s, *ω* = 12.57 rad/s [19]; and empty circle: μ* = 0.03 − 0.064*i* Pa*·*s, *ω* = 12.57 rad/s [19].

In Figure 3 we show how, in a viscous fluid, the maximum shear stress at the wall changes with fluid viscosity. This figure is equivalent to Figure A.2 in the paper by Abouali et al. [15]. Since the shear stress depends linearly on the viscosity of the fluid and also on the spatial derivatives of the velocity profile, predicting if the stress will increase or decrease with the viscosity is not obvious. In fact, Figures 2(a) and 2(b) show that as the viscosity decreases the derivative of the velocity at the wall increases. The results reported in Figure 3 show that the maximum shear stress at the wall increases nonlinearly with the viscosity and attains an asymptotic value for very viscous fluids. This maximum asymptotic value can be shown to be *A* *ρω* ^2^ *R* ^2^/5 (see also [15]). This implies that the adoption of high viscosity fluids as vitreous substitutes induces the generation of larger mechanical stresses on the retina. In the figure we report with vertical lines the cases corresponding to water and to two often used silicon oils (0.96 and 4.8 Pa*·*s) [17]. It appears that in the cases of the two oils the maximum stress on the retina is an order of magnitude higher than in the case of water. However, the differences between the two oils are small since, in both cases, the value of the maximum stress on retina is almost equal to the maximum possible asymptotic value.

Finally, we report in Figure 3 also points corresponding to the viscoelastic case, adopting for the rheological properties of the vitreous the values measured in [18, 19]. In these cases there is also an elastic component of the stress, the effect of which is to slightly increase the maximum wall shear stress with respect to the purely viscous case.

### 3.2. The Effect of the Existence of a Thin Layer of Aqueous between the Retina and the Vitreous Substitute

In the previous section we have discussed how the stress on the retina depends on the viscosity of a vitreous substitute, under the assumption that the fluid completely fills the vitreous chamber. In particular, we have shown that the mechanical actions on the retina grow with increasing fluid viscosity. In reality the situation is more complicated than this because, owing to the hydrophobic nature of vitreous substitutes, a thin layer of aqueous may form between the retina and the vitreous substitute.

We therefore now consider how the scenario described in the previous section is modified when we account for the presence of a thin layer of aqueous close to the retina.

**Figure 4:**
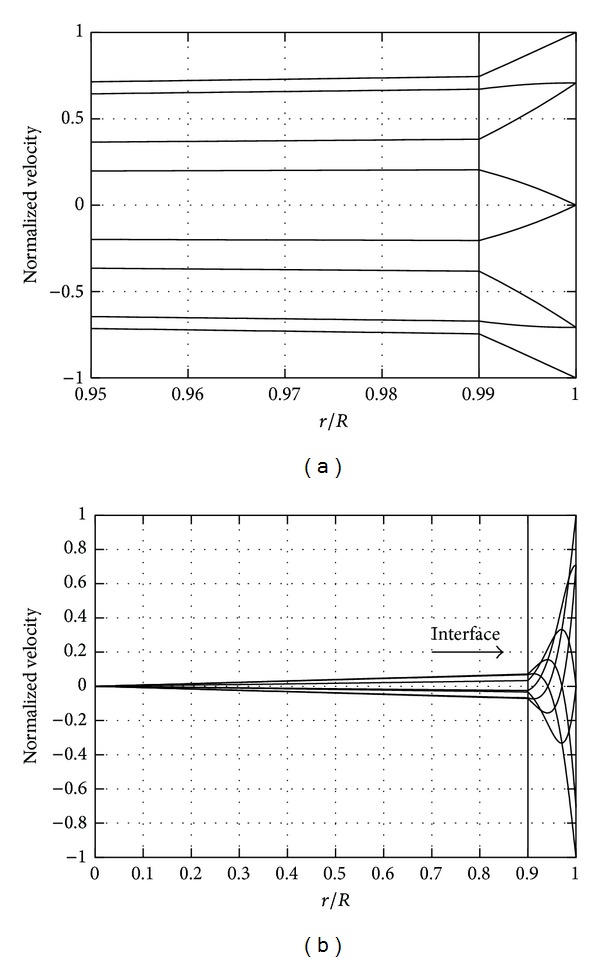
Velocity profiles in radial direction in the case in which the vitreous chamber contains two immiscible fluids.  *r* = 0 corresponds to the centre of the sphere and *r* = 1 corresponds to the location of the wall. The velocity is normalized with the maximum velocity at the wall. The frequency of rotations is equal to 10 rad/s. Vitreous substitute *μ* = 1 Pa*·*s; water, *μ* = 0.001 Pa*·*s. (a) *d* = 0.01*R* and (b) *d* = 0.1*R*.

In Figures 4(a) and 4(b) we show azimuthal velocity profiles on the equatorial plane at different times. The position of the interface between the two fluids is shown in the figure with a vertical solid line. The velocity profiles are continuous across the interface between the two fluids, but their slope is not. This is due to differences between the two fluids viscosities (we assumed in the figure *μ*_*a*_ = 10^−3^ Pa*·*s for the aqueous and *μ*_*vs*_ = 1 Pa*·*s for the vitreous substitute, e.g., a silicon oil). Figures 4(a) and 4(b) differ because a different thickness *d* of the aqueous layer has been assumed. In the first case (Figure 4(a)) we consider a thickness of the aqueous layer smaller than the thickness *δ* of the boundary layer that would form at the wall if the aqueous was completely filling the vitreous chamber (*d* < *δ*). In this case the motion of the wall is also felt in the vitreous substitute, which moves with a significant velocity. On the other hand, when *d* > *δ*, most of the motion keeps confined within the aqueous layer and the velocity in vitreous substitute is very small (Figure 4(b)). In other words in the latter case the vitreous substitute barely feels the motion of the wall.

**Figure 5:**
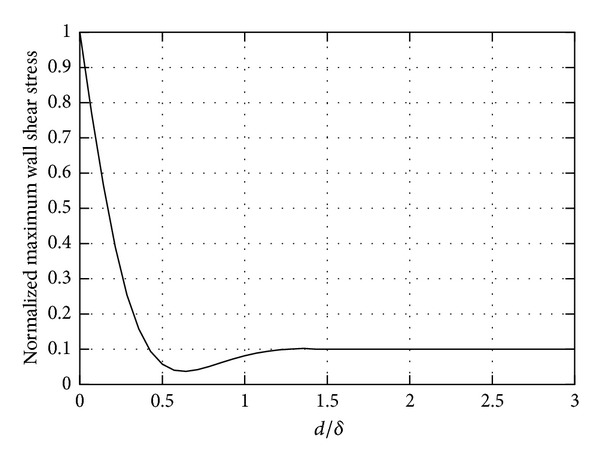
Maximum stress at the wall versus the thickness of the aqueous layer. The stress is normalized to 1, and the thickness of the layer *d* is scaled with *δ*, computed using the viscosity of water. Vitreous substitute *μ* = 0.96 Pa*·*s; water, *μ* = 0.001 Pa*·*s.

This has important implications for the wall shear stress at the wall, as it is shown in Figure 5. In the figure we plot the maximum stress at the wall versus the thickness of the aqueous layer. For the sake of clarity, we use dimensionless variables. The stress is normalized with the stress that would be obtained at the wall if the vitreous substitute was completely filling the domain. The thickness of the layer *d* is scaled with *δ*, computed as √(*μ*/*ρω*) and using the viscosity of the aqueous. When *d*/*δ* tends to zero, the scaled stress obviously tends to 1 (vitreous substitute alone) and the stress on the wall is maximum. However, the figure shows that it is sufficient for a thin layer of aqueous to be present to make the maximum shear stress at the wall drop significantly. When *d*/*δ* ≈ 1 or greater, the presence of the vitreous substitute is not felt by the wall and the stress drops to the value it would attain in the presence of aqueous alone. This simple model highlights the importance of accounting for the possible presence of the thin layer of aqueous at the wall in the calculation of the stress on the retina.

### 3.3. Stability of the Interface between Aqueous and Vitreous Substitute

The presence of an aqueous layer separating the vitreous substitute from the retina was shown in the previous section to have an important effect on the shear stress on the retina. It is also known that one of the main complications after injection of long-term vitreous replacement fluids (particularly silicon oils) is the possible occurrence of emulsification. This implies that the oil-aqueous interface might break, eventually leading to the formation of oil droplets dispersed in the aqueous. There are several possible causes of generation of an emulsion, with one of them being introduction of mechanical energy into the system that breaks down the oil aqueous interface [20]. Many authors have hypothesized that shear stresses at the tamponade fluid-aqueous interface generated during eye rotations play a crucial role in the generation of an emulsion [21, 22].

In order to investigate the feasibility of this assumption and determine which parameters play a role in the breakdown of the interface, we present in this section results from an idealized, yet informative, theoretical model. As discussed in Section 2 we assume that the aqueous layer in contact with the retina is much smaller than the radius of the eye and we neglect the curvature of the eye wall, treating the problem as two-dimensional (see Figure 1(c)). We perturb the flat configuration of the interface between the two fluids with a sinusoidal wave and investigate whether the amplitude of this wave grows or decays in time, with the aim of identifying threshold conditions for instability as the values of the controlling parameters are changed.

The problem of the stability of the interface is governed by the four dimensionless parameters introduced and described in the appendices. Here we discuss the role of two of them: *m* = *μ*_*vs*_/*μ*_*a*_, which is the ratio between the viscosities of the two fluids, and *S* = *σ*/(*ρdU* ^2^), which represents a dimensionless surface tension at the interface, where *σ* denotes the dimensional surface tension between the two fluids, *ρ* denotes fluid density, *d* is the thickness of the aqueous layer, and *U* is the maximum wall velocity. We note that, for the sake of simplicity, we neglect possible differences between the densities of the two fluids, thus effectively neglecting the role of gravity. The other dimensionless parameters that govern the stability problem are set to values that are reasonable for real eye rotations.

**Figure 6:**
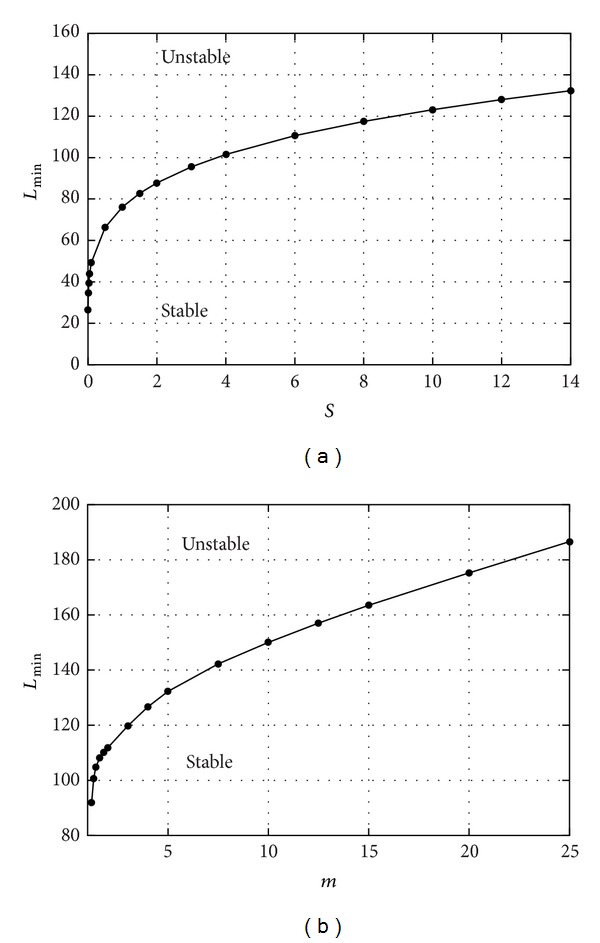
Length of the shortest unstable perturbation *L*_min⁡_, scaled with the thickness of the aqueous layer *d* versus *S* (a) and *m* (b). *R* = 12 and *ω* = 0.003 (*m* = 5 (a) and *S* = 14 (b)).

Our stability analysis shows that very long waves on the interface are invariably unstable during certain phases of the oscillation cycle. In other words the amplitude of very long disturbances always grows in time. We note that in the absence of an interface this stability problem consists in the stability of the so-called “Stokes boundary layer,” that is, the flow of a single fluid over an oscillating wall. This problem has been largely studied in the literature [23] and it is known to be stable in the range of parameters considered here. Therefore, we can conclude that the instability mechanism is indeed related to the existence of the interface. Very long waves might not be able to form within the eye globe, owing to the three-dimensionality of the domain (they will not effectively fit in the eye). Short waves, on the other hand, are stabilized by the surface tension acting on the interface. In Figures 6(a) and 6(b) we show how the length of the shortest unstable wave depends on the controlling parameters. In particular we focus on the role of the two dimensionless parameters *S* and *m*.

Figure 6(a) shows that, as the value of the (dimensionless) surface tension decreases, instability progressively affects shorter perturbations. This can be interpreted as follows. When the surface tension decreases, the interface effectively becomes more unstable, since even relatively short waves are predicted to be unstable and thus their amplitude is expected to grow in time. The stabilizing role of the surface tension too is not surprising in the light of results from stability analyses performed on similar problems [24].

In Figure 6(b) we show the effect of changing the ratio *m* between the viscosities of the two fluids. Note that the viscosity of silicon oils is much larger than that of water. The figure shows that as *m* increases the system becomes more stable, again meaning with this statement that only very long waves are expected to possibly grow in time. Conversely, for relatively small values of *m* progressively shorter waves are found to be unstable.

## 4. Conclusions

In the present paper we have discussed theoretical results from three different idealized mathematical models that, in our view, help in understanding some of the basic features of the fluid mechanics of vitreous substitutes in the eye. We have focused our attention on the flow generated in the vitreous chamber by rotations of the eye globe, which is by far the most important mechanism generating fluid motion.

We first have considered the case in which the whole vitreous chamber is filled with a single fluid and have modelled the chamber as a rigid sphere, performing sinusoidal small amplitude torsional oscillations, similar to what was done by previous authors [10, 11]. We have shown that, when the fluid is purely viscous, the maximum velocity is invariably attained at the sphere wall and the velocity at the centre of the domain is zero. In the limit of very large fluid viscosity the velocity profiles are approximately straight lines and the fluid moves almost as a rigid body. In the opposite limit of low viscosity, an oscillatory boundary layer forms at the wall and the fluid velocity in the core of the vitreous chamber is almost zero. We have shown that the maximum wall shear stress on the retina grows with increasing viscosity of the fluid in a highly nonlinear way and reaches an asymptotic value in the limit of high viscosity fluids, which is easily predicted analytically. This is relevant for the choice of vitreous replacement fluids. In fact the model shows that if the vitreous is replaced with a highly viscous fluid, mechanical actions of the retina should be expected to increase. This is, for instance, the case with silicon oils. In the clinical practice silicon oils with a viscosity of 1000 centistokes or 5000 centistokes are typically adopted. We remark that in both cases the viscosity is so large that the maximum values of the shear stress at the retina are close to its maximum possible asymptotic values. This means that, in terms of mechanical stresses on the retina, the two oils are equivalent to each other.

We have also briefly recalled how flow characteristics change when a viscoelastic fluid fills the vitreous chamber. The real healthy vitreous has viscoelastic properties, and there is a large body of research devoted to the identification of vitreous replacement fluids with viscoelastic properties. We have recalled that the motion of a viscoelastic fluid can be resonantly excited by eye rotations and, if this happens, large values of the shear stress are expected to develop on the retina. This has important implications for the choice of the ideal properties of vitreous substitutes. Soman and Banerjee [25] and Swindle and Ravi (2007) [26] review all materials currently in use, discuss their advantages and disadvantages, and list the characteristics of an ideal vitreous substitute. In their papers it is mentioned that the ideal substitute should have a large enough elastic component, so as to avoid excessive flow within the vitreous chamber. However, the possible occurrence of resonance as a risk factor for generating large mechanical stresses on the retina is disregarded.

In the second part of the paper we considered the effect of a thin layer of aqueous separating the vitreous substitute from the retina. Since vitreous substitutes are normally hydrophobic fluids and complete filling of the vitreous chamber can be hardly obtained, a layer of aqueous in correspondence with the retina is likely to form. We have shown that, when this is the case, the maximum stress on the retina can be significantly reduced, even if the viscosity of the vitreous replacement fluid is very large. Therefore, the possible existence of an aqueous layer should be accounted for when estimating the mechanical stresses on the retina after injection of a vitreous substitute.

The presence of an aqueous layer and, consequently, of an interface between the aqueous and the vitreous substitute also has a crucial effect in the possible development of an emulsion, which is one of the main drawbacks associated with the use of silicon oils. Making use of a simple mathematical model we have studied the stability of the aqueous-vitreous substitute interface. The results show that the interface becomes more unstable if the surface tension decreases and it becomes more stable if the viscosity of the vitreous substitute is higher. Both results are in agreement with clinical observations. In fact there is evidence that the tendency to emulsification is significantly enhanced by the presence of surfactants that decrease the surface tension between the two fluids [27]. Moreover, clinical experience shows that highly viscous vitreous substitutes are more resistant to emulsification than less viscous ones [28–30]. Obviously, our model only represents in a highly idealized fashion the real behaviour of the aqueous-vitreous substitute interface in the vitreous chamber during eye rotations and we are perfectly aware that reality is much more complex than we have assumed. However, to our best knowledge this is the first attempt to study the instability processes that might lead to the formation of an emulsion in the vitreous chamber and we believe that stability analyses such as the one proposed here can significantly contribute to highlighting the basic physical mechanisms taking place and to guiding the interpretation of more realistic models, as indeed it has been the case in many other physical contexts.

## Appendices

### A. Model 1

We consider a hollow rigid sphere with radius *R* performing periodic torsional oscillations of amplitude *A* and frequency *ω* about an axis passing through its centre (see Figure 1(a)). The angular displacement *β* of the sphere in time is described by the following time law:

(A.1) $$\begin{array}{c} \beta \left(t\right)=-A\cos\left(\omega t\right), \end{array}$$

with *t* time. We assume that the amplitude of oscillations is small (*A* ≪ 1).

The motion of a viscous fluid within the sphere is governed by the Navier-Stokes equations and the continuity equation, which read

(A.2a) $$\begin{array}{c} \frac{\partial u}{\partial t}+\left(u\cdot \nabla \right)u+\frac{1}{\rho }\nabla p-\frac{\mu }{\rho }\nabla^{2}u=0, \end{array}$$

(A.2b) $$\begin{array}{c} \nabla \cdot u=0, \end{array}$$

subject to the following boundary conditions:

(A.3a) $$\begin{array}{c} u=0\;\;\left(r=R\right) \end{array}$$

(A.3b) $$\begin{array}{c} v=0\;\;\left(r=R\right) \end{array}$$

(A.3c) $$\begin{array}{c} w=A\omega R\sin\left(\omega t\right)\;\;\left(r=R\right) \end{array}$$

(A.3d) $$\begin{array}{c} \text{regularity}\;\text{conditions}\;\;\left(r=0\right), \end{array}$$

where *u*, *v*, and *w* represent the radial, zenithal, and azimuthal components of the velocity, *p* is pressure, *ρ* is density, and *μ* is the dynamic viscosity of the fluid.

Taking advantage of the assumption of small amplitude eye rotations (*A* ≪ 1) the above equations can be linearized and solved in closed form. The velocity is purely azimuthal and the solution reads

(A.4) $$\begin{array}{c} u=v=0,\\ w=-\frac{iA\omega }{2}{\left(\frac{R}{r}\right)}^{2}\frac{R\sin\left(kr/R\right)-\mathrm{kr}\cos\left(kr/R\right)}{\sin k-k\cos k}e^{i\omega t}\sin\theta +\text{c}.\text{c}.,\\ p=\text{const}. \end{array}$$

In the above expression c.c. denotes the complex conjugate, *θ* is the zenithal coordinate (*θ* = 0; *π* identifies the axis of rotation), and

(A.5a) $$\begin{array}{c} k=\frac{\sqrt{2}}{2}\alpha \left(1-i\right), \end{array}$$

(A.5b) $$\begin{array}{c} \alpha =\sqrt{\frac{\rho \omega R^{2}}{\mu }}, \end{array}$$

where *α* is a dimensionless number named the Womersley number. The corresponding solution for the wall shear stress is

(A.6) $$\begin{array}{c} \tau =-\frac{\rho A}{2}{\left(\omega R\right)}^{2}\left(\frac{1}{1-k\text{cot}\;k}-\frac{3}{k^{2}}\right)\sin\theta e^{i\omega t}+\text{c}.\text{c}. \end{array}$$

and the maximum of *τ* is located on the equatorial plane *θ* = *π*/2. The maximum wall shear stress over a period of oscillation and over space grows with the fluid viscosity *ν* and reaches the following limiting value *τ*_max⁡_ as *ν* → *∞* (with *ν* = *μ*/*ρ* being the kinematic viscosity of the fluid):

(A.7) $$\begin{array}{c} \tau_{\max}=\frac{\rho A}{5}{\left(\omega R\right)}^{2}. \end{array}$$

The solution for the motion of a viscoelastic fluid is obtained by introducing a complex viscosity (i.e., a complex Womersley number in (A.5a)); see [10, 11] for further details.

### B. Model 2

We now take into account the presence of a thin layer of aqueous between the retina and the vitreous substitute fluid. We assume that the two fluids have the same density *ρ* but different viscosities (*μ*_*a*_ for the aqueous and *μ*_*vs*_ for the vitreous substitute). For the sake of simplicity we assume that the aqueous layer is arranged concentrically with respect to the vitreous substitute, as shown in Figure 1(b), so that the aqueous layer thickness is constant and equal to *d*.

The problem is still governed by the Navier-Stokes equations for the two fluids and, at the interface between the fluids, we impose the continuity of the velocity and the dynamic boundary condition. Assuming again that the sphere rotates according to (A.1) and that *A* ≪ 1 the solution can be found analytically and reads

(B.1a) ua=0

(B.1b) va=0

(B.1c) $$\begin{array}{c} u_{vs}=0 \end{array}$$

(B.1d) $$\begin{array}{c} v_{vs}=0 \end{array}$$

(B.1e) wvs=c1Aω(Rkvsr)2[Rsin⁡(kvsrR)−kvsrcos⁡⁡(kvsrR)] ×eiωtsin⁡θ+c.c.

(B.1f) wa=Aω(Rkar)2{c2[Rsin⁡(karR)−karcos⁡⁡(karR)]+c3[Rcos⁡⁡(karR)+karsin⁡(karR)]} ×eiωtsin⁡θ+c.c.,

where the subscripts *a* and *vs* denote the aqueous and the vitreous substitute, respectively. Moreover, the constants *c*_1_, *c*_2_, and *c*_3_ are determined by the boundary conditions and *k*_*a*_ and *k*_*vs*_ are given by (A.5a) and (A.5b) using the viscosity of the aqueous and the vitreous substitute, respectively.

The wall shear stress on the equatorial plane is equal to

(B.2) τ|θ=π/2=Aμaω[(1−3ka2)(c2sin⁡ka+c3cos⁡⁡ka)+3ka2(c2cos⁡⁡ka+c3sin⁡ka)]eiωt+c.c.

### C. Model 3

We now wish to study the stability of the interface between the aqueous layer and the vitreous substitute. For simplicity we assume that the thickness *d* of the aqueous layer is much smaller than the radius of the sphere *R* and, as a first approach to the problem, we neglect the effect of wall curvature and consider a two-dimensional problem in the (*x*, *y*) plane (see Figure 1(c)). Thus we consider two immiscible fluids occupying the regions of space 0 ≤ *y* < *d* and *y* > *d*, respectively, with kinematic viscosities *ν*_*a*_ and *ν*_*vs*_, and again assume that the two fluids have the same density. The flow is induced by periodic motion of the rigid wall, located at *y* = 0, with amplitude *A* and frequency *ω*.

We work in terms of the following dimensionless variables (denoted by superscript stars):

(C.1) $$\begin{array}{c} \left(x^{*},y^{*}\right)=\frac{\left(x,y\right)}{d},\;\;u_{i}^{*}=\frac{u_{i}}{U},\\ p_{i}^{*}=\frac{p_{i}}{\rho_{1}U^{2}},\;\;t^{*}=\frac{U}{d}t, \end{array}$$

where *U* is the maximum wall velocity and the subscript *i* denotes either the aqueous (*i* = *a*) or the vitreous substitute (*i* = *vs*). By scaling the governing equations we introduce the following dimensionless parameters:

(C.2a) $$\begin{array}{c} m=\frac{\mu_{vs}}{\mu_{a}}, \end{array}$$

(C.2b) $$\begin{array}{c} R=\frac{Ud}{\nu_{a}}, \end{array}$$

(C.2c) $$\begin{array}{c} S=\frac{\sigma }{\rho dU^{2}}, \end{array}$$

(C.2d) $$\begin{array}{c} \omega^{*}=\frac{d}{U}\omega , \end{array}$$

where *m* represents the ratio between the fluid kinematic viscosities, *R* is the Reynolds number of the flow (based on the aqueous viscosity), *S* is a dimensionless surface tension (where *σ* is the dimensional surface tension on the interface), and *ω** is a dimensionless frequency.

We decompose the flow in a basic state and infinitesimally small perturbation as follows:

(C.3a) $$\begin{array}{c} u_{i}^{*}=U_{i}^{*}+{\overset{-}{u}}_{i}^{*}, \end{array}$$

(C.3b) $$\begin{array}{c} p_{i}^{*}=P_{i}^{*}+{\overset{-}{p}}_{i}^{*}, \end{array}$$

where capital letters indicate the basic flow and small letters with a bar refer to perturbation quantities.

The basic flow is unidirectional (in the *x*-direction) and can be solved in closed form. We do not report the details here for the sake of space.

For the stability analysis we consider two-dimensional perturbations ${\overset{-}{u}}^{*}=({\overset{-}{u}}_{x}^{*},{\overset{-}{u}}_{y}^{*},0)$. This allows us to introduce the stream function *ψ*, defined as

(C.4a) $$\begin{array}{c} u_{xi}^{*}=\frac{\partial {\overset{-}{\psi }}_{i}}{\partial y}, \end{array}$$

(C.4b) $$\begin{array}{c} u_{yi}^{*}=-\frac{\partial {\overset{-}{\psi }}_{i}}{\partial x}. \end{array}$$

We adopt the quasi-steady approach; that is, we assume that perturbations evolve on a time scale that is much smaller than the characteristic time scale of the basic flow. This implies that we study the stability of a “frozen” basic flow at time *τ*, with 0 ≤ *τ* < 2*π*/*ω*. The suitability of this approach can be verified a posteriori by checking the relative magnitude of the time scale of perturbations with respect to that of the basic flow.

Taking advantage of the assumed infinite extension of the domain in the *x*-direction we expand the unknowns in Fourier modes as follows:

(C.5) $$\begin{array}{c} {\overset{-}{\psi }}_{i}=e^{i\alpha (x-\Omega t)}\psi_{i}\left(y,\tau \right)+\text{c}.\text{c}., \end{array}$$

where *α* is the dimensionless wavenumber and *Ω* denotes the complex eigenvalue of the system, whose real part represents the phase speed of perturbations and whose imaginary part represents the growth rate. Moreover, let ${\overset{-}{\eta }}^{*}$ denote the dimensionless perturbation of the interface position, measured in units of *d*. We impose that

(C.6) $$\begin{array}{c} {\overset{-}{\eta }}^{*}=\eta \left(t\right)e^{i\alpha (x-\Omega t)}+\text{c}.\text{c}. \end{array}$$

The final system of the equations for the perturbation evolution is given by two Orr-Sommerfeld equations, one for each fluid, together with suitable boundary conditions [31]. The system can be written as a generalized eigenvalue problem:

(C.7) $$\begin{array}{c} Av=\Omega Bv. \end{array}$$

If *Im*⁡(*Ω*) < 0, the system is linearly stable; if, on the other hand, *Im*⁡(*Ω*) > 0, then the system is linearly unstable. Zero values of the growth rate separate the space into stable and unstable subspaces. The system (C.7) is discretized employing a second-order finite-difference scheme with uniform spatial step and is efficiently solved using an inverse iteration algorithm.
